# Editorial: Healthy Healthcare: Empirical Occupational Health Research and Evidence-Based Practice

**DOI:** 10.3389/fpsyg.2020.02236

**Published:** 2020-10-16

**Authors:** Annet H. de Lange, Lise Tevik Løvseth, Kevin Rui-Han Teoh, Marit Christensen

**Affiliations:** ^1^Department of Work and Organizational Psychology, Open University, Heerlen, Netherlands; ^2^Department of Human Resource Management, HAN University of Applied Sciences, Nijmegen, Netherlands; ^3^Department of Psychology, Norwegian University of Science and Technology (NTNU), Trondheim, Norway; ^4^Hotel School of Management, University of Stavanger, Stavanger, Norway; ^5^Department of Psychiatry, St. Olavs University Hospital, Trondheim, Norway; ^6^Birkbeck, University of London, London, United Kingdom

**Keywords:** healthy healthcare, worker health, patient outcomes, research agenda, organizational practice

## Healthy Healthcare: Lessons Learned and A New Research Agenda for Occupational Health Psychology

Many countries within the European Union report significant difficulties in retaining and recruiting healthcare workers and are facing increasing levels of predicted staff shortages over the long term (European Commission, 2020). A substantial amount of scientific research from the past few decades points to the importance of organizational practices and the psychosocial design of jobs as ways of promoting the occupational health of healthcare workers (Løvseth and de Lange, 2020). These practices, along with healthy job design, can help sustain the availability and continuity, as well as appropriate levels of quality in the delivery of healthcare (Løvseth and de Lange, 2020). Despite these suggestions, recurrent data shows that occupational health-related disorders such as burnout and depression are continually increasing among healthcare workers worldwide (Herkes et al., 2019; Schot et al., 2019; Greenberg et al., 2020; Teoh et al., 2020b; Wang et al., 2020).

The challenge, therefore, lies in translating the knowledge and insights established by occupational health psychology into healthy practices that influence the design of jobs within healthcare organizations. Contemporary researchers in occupational health psychology are making strides in generating new knowledge that has the potential to improve the health and well-being of both healthcare workers and patients (Robert et al., 2011; Teoh et al., 2019). However, this knowledge typically focuses on the work-related predictors and outcomes of healthcare workers and may not reach its full potential or be perceived as relevant problems to other relevant groups, including clinicians, leaders, or patients. This is because it often ignores indicators of patient care, and might exclude the influence of organizational practices or the wider system. As a discipline, occupational health psychology can do more to recognize the complexity of organizations, synergies, processes, and the relevance of context when developing knowledge related to healthcare organizations.

Current developments and challenges in healthcare create the need to develop new research agenda for occupational health psychology that emphasize the investigation of integrative perspectives, linking worker health and well-being to quality of patient care and the organization of healthcare services. The aim of this special issue, on the topic “*Healthy Healthcare*,” was to call for new occupational health psychology to develop research approaches and transfer evidence-based knowledge and practice to healthcare settings and its management (Løvseth and de Lange, 2020). Approaching occupational health psychology from a *Healthy Healthcare* perspective is important to generating new knowledge on the necessary pathways or interventions that could retain healthcare workers, and to maintain or positively influence the quality of healthcare service delivery.

This editorial, therefore, aims to: (i) introduce the concept of *Healthy Healthcare* and how it relates to occupational health psychology; (ii) summarize the accepted papers in this special issue and discuss how they relate to the concept of *Healthy Healthcare*; and (iii) to present a new research agenda, drawing on occupational health psychology research to further advance our understanding of the concept of *Healthy Healthcare*.

### Healthy Healthcare: A New Paradigm

“*Healthy Healthcare*” refers to a new interdisciplinary system-based perspective of healthcare practices encompassing three main pillars: (1) quality of patient care; (2) worker health and well-being; and (3) the organization and practices of healthcare organizations. It recognizes that healthcare systems must be organized, managed, and financed in balance with the health and performance of available workers in mind (Løvseth and de Lange, 2020). Moreover, it emphasizes the importance of a contingent perspective where one size does not fit all contexts and the heterogeneous workforce. This means that knowledge production within a *Healthy Healthcare* perspective should be sensitive to contextual factors and the continuous adaptation and changes in healthcare to meet societal developments. It also realizes that benefits in one pillar (e.g., patient care, workers health, organizational practice) can potentially disadvantage another pillar. Ultimately a system-based perspective considering the dynamics between the patient(s), the worker(s), and the complex healthcare system will lead to a more resource-efficient delivery of high-quality healthcare services.

Within this position paper, we focus on occupational health psychology as a discipline from which research and practice are crucial to inform and advance *Healthy Healthcare*. The inter-disciplinary nature of *Healthy Healthcare* aligns well with the discipline of occupational health psychology given that the latter is also inherently multidisciplinary and draws on occupational health and psychology as well as being inclusive of public health, human factors, organizational studies, economics, industrial engineering, and more (Houdmont and Leka, 2010). Crucially, the general principles of occupational health psychology (Cox et al., 2000) are that it is (a) an applied science, (b) evidence-driven, (c) oriented toward problem-solving, (d) multidisciplinary, (e) participatory, and (f) focused on intervention, with an emphasis on primary prevention, all of which resonate strongly with the concept of *Healthy Healthcare*.

## The Current Issue

The complexity of a system-based perspective of *Healthy Healthcare* requires a continuously interdisciplinary focus that is sensitive to contextual differences in healthcare practice. It also requires a variety of methodologies to study system components, their interrelation, the uniqueness of those relations, and their potential effects on each *Healthy Healthcare* pillar. To facilitate knowledge development about *Healthy Healthcare* from the perspective of occupational health psychology, this special issue called for new empirical as well as review studies in different contexts of healthcare that help to bridge understanding across the three *Healthy Healthcare* pillars: (i) the organization of healthcare; (ii) workers' health and well-being; and (iii) the quality of care provided.

In total, six papers were accepted. The special issue includes a systematic review examining the influence of psychosocial work characteristics in explaining the mental health of nursing staff (Broetje et al.). It also includes two different two-wave longitudinal panel studies examining age-related factors among aging healthcare workers (de Lange et al.; Van der Heijden et al.). There is a cross-sectional study investigating the relationship between job autonomy, self-leadership, work engagement, and health among healthcare workers (van Dorssen-Boog et al.), and a process-evaluation qualitative study among hospital executives about the key process factors in implementing health-related work design interventions (Genrich et al.). The issue also includes a qualitative study exploring the emerging psychosocial risks of healthcare accreditation in workplaces (Alshamsi et al.).

Together, these six papers offer important contributions, examining the relationships between each of *Healthy Healthcare* pillars (such as the relations between organizational practices, job design, and worker well-being) for different types of healthcare practices and contexts among a variety of healthcare workers, but also includes insights about the interrelation of the main pillars from the perspective of current systems. This includes healthcare assistants, nursing workers, upper and middle managers within a hospital, different levels of seniority as well as different levels of organizational practices. Moreover, the research questions of these papers address diverse issues related to *Healthy Healthcare* through different theoretical frameworks such as the JD-R Model and theory (Bakker and Demerouti, 2017), the Self-Determination Theory (Deci et al., 2017), Ajzen's Theory of Planned Behavior (Ajzen, 1991), the Selection Optimization and Compensation Theory (Baltes and Baltes, 1990), and the Socio-emotional Selectivity Theory (Carstensen, 2019).

These papers also contribute to *Healthy Healthcare* by using different methodological approaches, including qualitative and quantitative, cross-sectional and longitudinal, as well as a meta-analytical review approach. By using these different methodologies the papers provide valuable new in-depth insights into the mechanisms and processes within different aspects of *Healthy Healthcare*, including the importance of supportive work environments as well as healthy job design to create resourceful and healthy healthcare workers. In other words, these papers individually provide us with relevant new insights that enable us to further summarize the lessons learned and discuss unresolved issues of *Healthy Healthcare* as a concept.

### Lessons Learned and Unresolved Issues

Congruent with the majority of studies within occupational health psychology that focus on the healthcare sector, most articles in this special issue focus on only two out of the three pillars in the system perspective of *Healthy Healthcare*. The relationship between the organization of healthcare and worker efficiency and health or well-being. The effect on patient outcomes, such as indicators of patient safety, satisfaction, or other relevant patient-based outcomes is less frequently investigated. Existing research efforts could also benefit from a stronger emphasis on positive outcomes like work engagement or meaning of work, or the simultaneous interplay between positive and negative factors and outcomes in the different pillars of *Healthy Healthcare*; rather than the current main focus of existing research, which often considers the negative concepts of work demands and the unhealthy consequences of this for the workforce. Similarly, there is a need for more team-based or organizational-level outcomes, as individual-level data outcomes have tended to dominate research to date.

Studies that examine the relationship between all three *Healthy Healthcare* pillars are rare and could be facilitated by an interdisciplinary focus between occupational health psychology and, for instance, health economics, technology, or medicine. These are all contributing factors that hinder the uptake and implementation of knowledge gained from occupational health psychology in healthcare practices by administrators and policymakers. As these stakeholders are typically tasked with the delivery of resource-efficient systems and focussed on the quality of healthcare delivery, concepts related to technology in healthcare (Iyer et al., 2020), capacity planning (Gheasi and de Lange, 2020), clinical and economical concepts (Gheasi and de Lange, 2020) are particularly salient to them. These alternative perspectives and research approaches will help facilitate the uptake of evidence-based knowledge and practices from occupational health psychology into *Healthy Healthcare* practices that are fundamentally important for the development of the resource-efficient delivery of high-quality healthcare services by a competent, motivated, and healthy workforce.

## Healthy Healthcare: Research Agenda

One of the most important conclusions of the current issue is that these studies recognize the importance of sharing insights related to creating a concept of *Healthy Healthcare*. They identify and provide knowledge about ideas within each pillar and the interrelated aspects of the main pillars of the current system.

**Figure 1:**
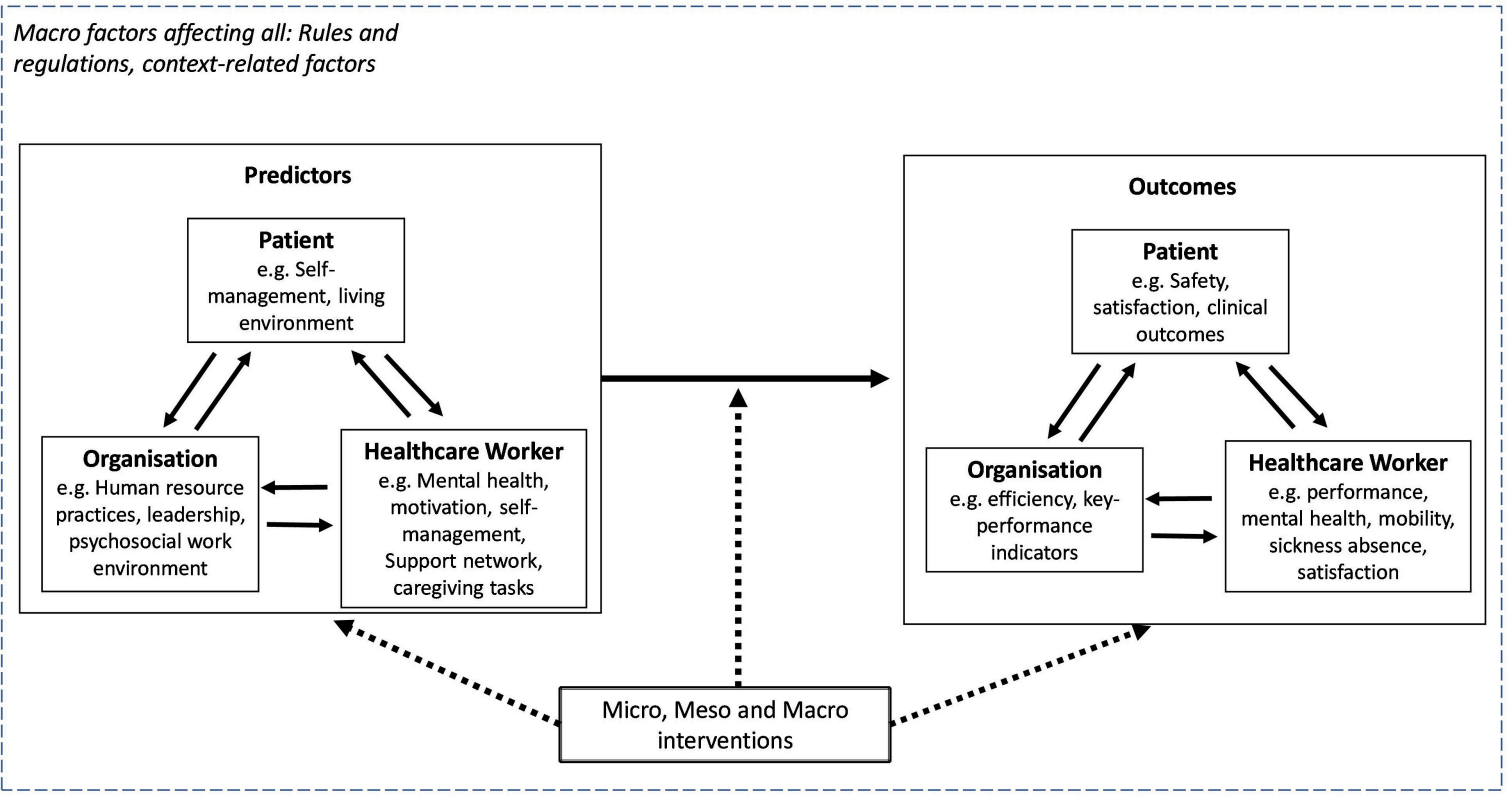
Possible research pathways Healthy Healthcare: factors at micro-, meso-, and macrolevel.

Taking up the system-based perspectives of *Healthy Healthcare* (Løvseth and de Lange, 2020), we present an updated integrative research model that can be used in future research of occupational health psychology (Figure 1). The model includes current pathways among occupational health psychology-related concepts and their outcomes at micro, meso, and macro levels. The model demonstrates the contextual sensitivity of this system-perspective at the individual level as well as within a wider societal, national, governmental, and macro context that influences all factors and relationships within the model (Teoh and Hassard, 2019; Gheasi and de Lange, 2020).

Based on the important contributions from the studies in this special issue, we have developed a *Healthy Healthcare* system perspective and model (Figure 1). We recommend that future research initiatives in occupational health psychology should consider:

1. *Developing studies and new overarching theories based on the system-perspective of Healthy Healthcare*. Although the topics included in this issue investigated one or two relevant pillars of *Healthy Healthcare* (e.g., mostly healthcare worker and organization), the full concept of *Healthy Healthcare* remains theoretically as well as empirically untested, and further research is needed to develop and examine the core concepts postulated by it. Emphasis needs to be placed on linking different antecedents of its three core pillars, including the mechanisms that explain these relationships to possible outcomes among patients, healthcare workers, and organizations. These will contribute to developing and refining the overarching theoretical model presented in Figure 1 above.
2. *Multilevel study designs*. Occupational health researchers typically neglect the fact that relationships are situated within a wider context, with important factors at the organizational, sectoral, societal, and national level all influencing the three pillars of *Healthy Healthcare* (Teoh and Hassard, 2019). Furthermore, factors at the individual level can influence macro-level outcomes (e.g., mortality and infection rates, patient satisfaction). The proliferation of more advanced multilevel analysis techniques and the collection of data across different levels and sources provides opportunities for researchers to capture the complexity of this system perspective within their study designs (Teoh et al., 2020a). The input of large-scale datasets on healthcare on a regional as well as a national level also offers new research directions.
3. *Capturing the diversity of the healthcare workforce*. Much of the existing research focuses on healthcare professionals, neglecting a large proportion of other workers in healthcare such as healthcare assistants, paramedics, administrators, porters, and in particular, unpaid workers (Clancy et al., 2019; see for exception de Lange et al. including supportive staff).

Besides, studies of diversity in terms of age (Van der Heijden et al.; de Lange et al.), gender, ethnicity, and immigrant workers (Mackert et al., 2011), studies of healthcare workers in developing and third-world countries (McCoy et al., 2008) are also less common. This is concerning, as unpaid workers are a large part of healthcare service delivery worldwide (Taylor, 2004) and an aging workforce implies demographic changes that substantially affect healthcare practice. Equally, ethnic minorities are more likely to experience poorer working conditions (Kinman et al., 2020), and that the gendered nature of healthcare work has implications for work-life boundaries among workers (Halford et al., 2015). A more inclusive and sustainable view of the workforce is needed to more accurately, and fairly, represent those working in the sector.

4. *Situating leadership within Healthy Healthcare*. The importance of leadership in creating healthy workplaces has been highlighted in earlier research (Furunes et al., 2018; Furunes, 2020), but a concept of health-promoting leadership has not yet been well established in occupational health research and models, which, therefore, warrants further exploration and new research. With critical questions being posed on how we can better understand the influence leadership has on the three *Healthy Healthcare* pillar—workers well-being (Nielsen and Taris, 2019), patient safety and care (Sfantou et al., 2017), and organizational systems and strategy (Bonardi et al., 2019)—developments here will have direct relevance for *Healthy Healthcare*, particularly where research looks at more than one pillar.
5. *Positive well-being*. The more detailed and holistic examination of what well-being is in the field of occupational health psychology has not yet caught-on within research involving the health services (Bakker et al., 2008; Scheepers et al., 2015). Here, the emphasis still is on ill-health and in particular, burnout. However, well-being exists as a much broader construct (Teoh et al., 2020a), and the narrative within related-healthcare research needs to shift to include more positive manifestations of well-being, including prevalence, their processes, and nomological networks, along with interventions. Crucially, this also encompasses patient care, with quality of care not merely being about the absence of disease or infirmity, but facilitating conditions that allow patients and society to thrive and flourish as well.
6. *Primary-interventions*. Within occupational health psychology, there has recently been a focus on the need to identify resources at multiple levels, which has called for interventions to strengthen resources at four levels within the organization: the Individual, the Group, the Leader, and the Organizational level (IGLO model). These potential interventions aim to ensure employee health and well-being (Day and Nielsen, 2017; Nielsen et al., 2017). The systems perspective embraced by *Healthy Healthcare* necessitates organizational-level participatory interventions. Much of the intervention research within healthcare has typically been at the individual level in the form of well-being (Regehr et al., 2014) or skills and competency-based training (Ginsburg et al., 2005). Where organizational-level interventions have been carried out (Weigl et al., 2013; Dixon-Woods et al., 2014), they have tended to focus only on two of the three pillars of *Healthy Healthcare*. Occupational health psychology can contribute to this, as it has seen exponential growth in our understanding of primary and organizational type interventions. Principles such as risk assessments, participation, manager support, and a continuous learning cycle are essential in this process, and more research is needed to support primary and multilevel interventions that seek to change the larger healthcare system (Nielsen and Noblet, 2018).
7. *Embrace different research methodologies and paradigms*. For all that a positivist paradigm can provide in establishing patterns and relationships, what are the work experience and processes that underpin *Healthy Healthcare*? While qualitative methods can explore some of these experiences, specific in-depth approaches (e.g., Interpretative Phenomenological Analysis) can also give voice and provide insights on how individuals make sense of the healthcare system (Peat et al., 2019). Equally, realist evaluation (such as the Context-Mechanism-Outcome framework) and process evaluation (Salter and Kothari, 2014; Nielsen and Miraglia, 2017) are pivotal to understand what worked for who and in what circumstances when it comes to knowledge transfer and interventions. Consequently, researchers need to embrace a wider range of paradigms and methods to better examine the concept of *Healthy Healthcare*.

## Conclusion

A system-based perspective is needed to address the challenges faced in healthcare and to increase the uptake of knowledge from occupational health psychology into healthcare. The *Healthy Healthcare* perspective provides a framework to do so by advocating for the examination and linking of three pillars for organizational practices, workers' health and well-being, and quality of patient care. Here, occupational health psychology is not only well placed to embrace *Healthy Healthcare*, but equally, offers considerable expertise and insights to advance the concept further. While the papers in this special issue shed important light in our understanding and the concepts of occupational health psychology, seven further points could also contribute to new future research agenda, namely: (i) developing an overarching theory and concepts of *Healthy Healthcare* (see the suggested framework in Figure 1); (ii) embracing more multi-level study designs; (iii) capturing the diversity of the healthcare workforce; (iv) situating leadership within *Healthy Healthcare*; (v) expanding our focus of well-being to include more positive manifestations; (vi) focusing on primary and organizational-level interventions; and (vii) embrac different research methodologies and paradigms.

## Author Contributions

All authors listed have made a substantial, direct and intellectual contribution to the work, and approved it for publication.

## Conflict of Interest

The authors declare that the research was conducted in the absence of any commercial or financial relationships that could be construed as a potential conflict of interest.
